# Effects of Prenatal Exposure to Pollutants on Children’s Development: Tang and Perera Respond

**DOI:** 10.1289/ehp.11763R

**Published:** 2008-10

**Authors:** Deliang Tang, Frederica P. Perera

**Affiliations:** Department of Environmental Health Sciences, Columbia University, New York, New York, E-mail: dt14@columbia.edu; Columbia Center for Children’s Environmental Health, Columbia University, New York, New York

The study we reported in our article “Effects of Prenatal Exposure to Coal-burning Pollutants on Children’s Development in China” (Tang et al. 2008) was conducted in the Tongliang County of Chongqing City, an area in western China. In his letter, Dórea suggests that the children who participated in our study may have been exposed to thimerosal in vaccinations. Because the actual levels of thimerosal used in each vaccine are a trade secret, we were not able to directly control for possible exposure to thimerosal in our analysis. We note that in the United States, all routinely recommended vaccines for U.S. infants are available only as thimerosal-free formulations or contain only trace amounts of thimerosal. We do not know whether that has been the case for vaccines used in China. On the other hand, the children’s vaccination schedule is well enforced in China, so any thimerosal exposure would have been similar among all the children who participated in our study. Therefore, this exposure would not have been likely to confound the observed relationship between Gesell Developmental scores (GDS) and PAH–DNA adducts and lead concentrations in newborn umbilical cord blood.

We did not observe a significant adverse impact of the Hg levels in cord blood on neurodevelopment at 2 years of age. The main concern for mercury exposure and neurodevelopment concerns prenatal exposures to methylmercury in fish consumed by pregnant women, and the consumption of fish is low in Tongliang. Reports have shown high Hg levels in rice from a mining area of Guizhou, a province located in southwestern China (Qiu et al. 2008; Feng et al. 2008). The Hg levels in the rice from other areas in China did not seem to exceed normal levels (Zhang and Wang 2007). Our study area, Tongliang County, is not within the Hg mining areas of Guizhou Province; thus, it is unlikely that the consumption of rice could expose the study sample to notable Hg levels.

In reference to Dórea’s suggestion to account for breast-feeding, we did incorporate a breast-feeding variable in our initial analysis. However, because the effect of breast-feeding was not generally statistically significant, the final model did not include a breastfeeding variable.

## References

[b1-ehp-116-a418b] Feng X, Li P, Qiu G, Wang S, Li G, Shang L (2008). Human exposure to methylmercury through rice intake in mercury mining areas, Guizhou province, China. Environ Sci Technol.

[b2-ehp-116-a418b] Qiu G, Feng X, Li P, Wang S, Li G, Shang L (2008). Methylmercury accumulation in rice *(Oryza sativ*a L.) grown at abandoned mercury mines in Guizhou, China. J Agric Food Chem.

[b3-ehp-116-a418b] Tang D, Li TY, Liu JJ, Zhou ZJ, Yuan T, Chen YH (2008). Effects of prenatal exposure to coal-burning pollutants on children’s development in China. Environ Health Perspect.

[b4-ehp-116-a418b] Zhang L, Wong MH (2007). Environmental mercury contamination in China: sources and impacts. Environ Int.

